# Fibulin2: a negative regulator of BMSC osteogenic differentiation in infected bone fracture healing

**DOI:** 10.1038/s12276-023-00942-0

**Published:** 2023-02-17

**Authors:** Shi-Dan Li, Wei Xing, Shao-Chuan Wang, You-Bin Li, Hao Jiang, Han-Xuan Zheng, Xiao-Ming Li, Jing Yang, De-Bin Guo, Xiao-Yu Xie, Ren-Qing Jiang, Chao Fan, Lei Li, Xiang Xu, Jun Fei

**Affiliations:** 1 Department of Orthopaedics, State Key Laboratory of Trauma, Burn and Combined Injury, Daping Hospital, Army Medical University, Chongqing, 400042 People’s Republic of China; 2 Department of Stem Cell and Regenerative Medicine, State Key Laboratory of Trauma, Burn and Combined Injury, Daping Hospital, Army Medical University, Chongqing, 400042 People’s Republic of China; 3 Department of Orthopaedics, Affiliated Hospital of Southwest Medical University, Luzhou, 646000 People’s Republic of China; 4 Department of Nursing, Montreal Neurological Hospital, 3801 Rue University, Montréal, QC H3A 2B4 Canada; 5 Department of Military Traffic Injury Prevention, Daping Hospital, Army Medical University, Chongqing, 400042 People’s Republic of China; 6 Department of Emergency, State Key Laboratory of Trauma, Burn and Combined Injury, Daping Hospital, Army Medical University, Chongqing, 400042 People’s Republic of China; 7 Department for Combat Casualty Care Training, Training Base for Army Health Care, Third Military Medical University, Chongqing, 400042 People’s Republic of China; 8 Medical Research Center, The Third People’s Hospital of Chengdu, Affiliated Hospital of Southwest Jiaotong University, Southwest Jiaotong University, Chengdu, 610031 People’s Republic of China

**Keywords:** Mesenchymal stem cells, Mechanisms of disease

## Abstract

Bone fracture remains a common occurrence, with a population-weighted incidence of approximately 3.21 per 1000. In addition, approximately 2% to 50% of patients with skeletal fractures will develop an infection, one of the causes of disordered bone healing. Dysfunction of bone marrow mesenchymal stem cells (BMSCs) plays a key role in disordered bone repair. However, the specific mechanisms underlying BMSC dysfunction caused by bone infection are largely unknown. In this study, we discovered that Fibulin2 expression was upregulated in infected bone tissues and that BMSCs were the source of infection-induced Fibulin2. Importantly, Fibulin2 knockout accelerated mineralized bone formation during skeletal development and inhibited inflammatory bone resorption. We demonstrated that Fibulin2 suppressed BMSC osteogenic differentiation by binding to Notch2 and inactivating the Notch2 signaling pathway. Moreover, Fibulin2 knockdown restored Notch2 pathway activation and promoted BMSC osteogenesis; these outcomes were abolished by DAPT, a Notch inhibitor. Furthermore, transplanted Fibulin2 knockdown BMSCs displayed better bone repair potential in vivo. Altogether, Fibulin2 is a negative regulator of BMSC osteogenic differentiation that inhibits osteogenesis by inactivating the Notch2 signaling pathway in infected bone.

## Bone fracture: Identifying a protein that hinders repair during infection

A protein that is overexpressed during bone fracture infection blocks a key signaling pathway and prevents bone repair. Infection following bone fracture remains a leading cause of delayed or unsuccessful healing. Stem cells from bone marrow are the main source of osteoblasts, bone-forming cells necessary for healthy bone regeneration and repair. Infection causes disruption to stem cell function and hinders bone healing, but the exact mechanisms involved remain unclear. In experiments on human tissue samples and a mouse model, Jun Fei and Xiang Xu at the Army Medical University in Chongqing, China, and co-workers examined the role of the fibulin2 protein in infected bone fractures. Fibulin2 is overexpressed in infected tissues, and the protein suppresses stem cell differentiation into osteoblasts by deactivating a key signaling pathway. Blocking fibulin2 in mice models restored bone formation.

## Introduction

As a mineralized organ, bone is indispensable for load bearing and movement of the body^1^. Bone fracture occurs when cortical bone continuity is impaired and is characterized by localized swelling, severe pain and motor dysfunction^2^. As society develops, bone fracture becomes a major drain on health care resources^3^. Notably, approximately 7.9 million fractures are reported annually, costing $21 billion in the United States alone^4^. The population-weighted incidence rate of fracture was approximately 3.21 per 1000 in China^5^. Theoretically, fractured bone can self-repair and regenerate via intramembranous and endochondral ossification, in which bone tissue is woven and precursor cells differentiate into osteoblasts in a callus^6^. However, in approximately 10% of fractures, union is delayed or unsuccessful due to unstable fixation, blood supply interruptions, or, in most cases, the presence of infection^7–9^. There is an urgent medical need to develop new strategies to promote bone healing and prevent delayed union or nonunion.

Previous research has revealed that the incidence of infection following skeletal fracture ranges from 2% to 50%^10^. The onset of infection can lead to delayed skeletal union or nonunion, ultimately causing bone defects^11,12^. Bone is a dynamic organ that undergoes lifelong remodeling regulated by the relative equilibrium between bone formation mediated by the mesenchymal stem cell–osteoblast lineage and bone resorption mediated by the monocyte–macrophage–osteoclast lineage^13,14^. Infection disrupts this reciprocal balance, enhancing bone resorption and slowing bone formation, but the underlying mechanisms are unclear^15^. For instance, James et al. demonstrated that infection triggered osteoblast death, and Ayelén et al. showed that infection modulated osteoclastogenesis; both of these outcomes are detrimental to bone healing^11,16^.

Buried within bone, bone marrow mesenchymal stem cells (BMSCs) are the major progenitors of osteoblasts^17^, which are important for bone repair and regeneration^18^. Therefore, BMSCs are widely used in tissue engineering-based bone regenerative strategies^19^. As described in previous reports, microorganisms can induce cytokine production in infected bone tissue, which can lead to BMSC dysfunction and thus disrupt bone healing^20^. To the best of our knowledge, the specific mechanisms underlying BMSC dysfunction-mediated bone destruction in the context of infection are largely unknown. Therefore, a clear understanding of the effect of bone infection on BMSC function and related regulatory mechanisms is essential for preventing delayed union or nonunion and promoting bone fracture healing^14^.

In our previous study, we found that Fibulin2 protein expression was upregulated in the plasma after infection^21^. Furthermore, proteomic analysis revealed that Fibulin2 expression was elevated in infected bone. Hence, we speculated that Fibulin2 is involved in delayed bone union or nonunion caused by bone infection. Fibulin2, a 180-kD protein, was first identified in 1990 by Kluge et al. ^22,23^. It contains multiple calcium-binding sites in a tandem array of 11 epidermal growth factor (EGF)-like motifs^24^. EGF-like domains are pervasive in many proteins, such as Notch and its ligands, laminin, fibulin, TGF-α and coagulation factors^25^. A recent study provided evidence that certain proteins with EGF-like motifs may interact with Notch^26^. In addition, Hu et al. ^27^ reported that Fibulin3 may activate Notch signaling. Finally, the canonical Notch pathway clearly affects osteogenesis^28^. Based on these findings, we hypothesized that Fibulin2 may be a negative regulator that interacts with the Notch signaling pathway and inhibits bone remodeling during infected bone fracture healing.

In this study, Fibulin2 was discovered to be closely associated with bone infection. Elevated Fibulin2 expression was found in BMSCs following bacterial component stimulation. We also confirmed that higher Fibulin2 levels led to weakened BMSC osteogenic differentiation; this effect was mediated through the Notch2 pathway. Furthermore, Fibulin2 knockout (*Fibulin2−/−*) accelerated mineralized bone formation during skeletal development and reduced inflammatory bone loss in adult mice with infection. In particular, our study sheds light on the mechanism of bone disorders originating from bone infection and suggests a novel target for promising drugs to prevent delayed union or nonunion and promote bone healing.

## Materials and methods

### Collection of human bone tissue and bone marrow

We gathered bone specimens from patients with or without bone infection. Bone marrow was collected from patients with osteonecrosis of the femoral head (ONFH) during therapeutic surgery. Ten bone specimens were included in our proteomic analysis, and five other pathological bone specimens were subjected to sectioning. In addition, BMSCs were harvested from the bone marrow of three volunteers (Supplementary Table 1).

### Proteomics analyses

Proteomics analyses were performed at Sinotech Genomics Inc. (Shanghai, China) according to a standard procedure. Briefly, bone tissues were ground into powder in liquid nitrogen and suspended in lysis buffer (1% sodium deoxycholate (SDS) and 8 M urea) to extract total protein. Then, the extracted protein was quantified, qualified, and digested. The resulting peptide mixture was labeled using 10-plex tandem mass tag (TMT) reagent (Thermo Fisher, USA). Next, the samples were pooled and fractionated by ACQUITY ultrahigh-performance liquid chromatography (Waters, USA) on an ACQUITY UPLC BEH C18 column (1.7 µm, 2.1 mm × 150 mm, Waters, USA) to increase the proteomic depth. Labeled peptides were analyzed by online nanoflow liquid chromatography with tandem mass spectrometry on a 9RKFSG2_NCS-3500R system (Thermo Fisher, USA) connected to a Q Exactive HF-X (Thermo Fisher, USA) with a nanoelectrospray ion source. Finally, the raw data were analyzed to identify differentially expressed proteins at a false discovery rate (FDR) cutoff of 0.05 (95% confidence interval).

### Histology and immunohistochemistry

Bone samples were fixed with 4% paraformaldehyde for 24 h, decalcified with 15% EDTA for 2 weeks, processed into paraffin sections at a thickness of 4 μm, and subjected to HE and Masson’s trichrome staining. Immunohistochemistry to detect Fibulin2, Notch2, and Runx2 on the sections was performed using primary antibodies against Fibulin2 (Abcam, ab234993, 1:250), Notch2 (CST, 57325, 1:100), and Runx2 (Abcam, ab192256, 1:250). The stained sections were scanned under a microscope (Leica, Germany).

### Isolation of BMSCs

BMSCs were isolated from bone marrow obtained from discarded tissue during hip arthroplasty. Heparinized human bone marrow was mixed with serum-free stem cell culture medium (VivaCell, Shanghai, China) containing an equal proportion of 4% human platelet lysate (VivaCell, Shanghai, China). Then, the mixture was incubated in a humidified atmosphere containing 5% CO_2_ at 37 °C. After seven days, we began refreshing the medium every 3 days. Once the cells reached 80% confluence, P0 cells were harvested. Several antibodies, including FITC-conjugated anti-CD90, PerCP-Cy™5.5-conjugated anti-CD105 and allophycocyanin (APC)-conjugated anti-CD73 and phycoerythrin (PE)-conjugated antibodies against CD45, CD34, CD11b, CD19 and HLA-DR (BD Biosciences, USA), were used for flow cytometry-based phenotypic cell identification. Osteogenic, adipogenic and chondrogenic differentiation assays were performed to characterize the multidifferentiation potential of the BMSCs.

### Osteoblastic differentiation and treatment

For analysis of osteogenic differentiation induced by Fibulin2, human BMSCs were seeded into a 6-well plate (1 × 10^5^ cells per well) and cultured in MSC rapid osteogenic differentiation medium (Biological Industries, Israel) containing Fibulin2 or control (PBS). The medium was changed every 2–3 days. After 5 or 10 days of incubation, the cells were harvested for RT–qPCR and western blotting (WB). On Day 5, Alp staining was performed using an Alp detection kit (Beijing Solarbio Science & Technology Co., Ltd.) according to the manufacturer’s protocol. On Day 10, the cells were subjected to ARS with an AR detection kit (ScienceCell, USA) to visualize mineralized nodule formation.

### Immunofluorescence

BMSCs were seeded on glass slides, fixed with 4% paraformaldehyde for 20 min, blocked in PBS with 5% goat serum for 1 h, and then stained overnight with rabbit anti-Notch2 antibody (CST, 4530T, 1:400) and mouse anti-Fibulin2 antibody (Santa Cruz Biotechnology, sc-271263, 1:100). Goat anti-rabbit Cy3 secondary antibody (Beyotime, A0516, 1:500) and goat anti-mouse Alexa Fluor 488 secondary antibody (Beyotime, A0423, 1:500) were used. Images were counterstained with DAPI (Sigma, D8417) and acquired with a confocal microscope (Leica).

### Transfection of lentiviral constructs

shRNA against Fibulin2 was used to establish sh-FBLN2 in a GV493 lentiviral vector. Full-length FBLN2 was cloned into a GV640 lentiviral vector. All lentiviruses and respective negative controls were synthesized by GeneChem (Shanghai, China). BMSCs were cultured in 25 cm^2^ monolayers in culture medium to a density of approximately 5000 cells per cm^2^. The cells were transfected at a multiplicity of infection of 10 with lentiviral particles expressing shRNA directed against the Fibulin2 transcript (Shanghai GeneChem Co.), nontargeting control shRNA (scramble), or an unloaded control (vehicle) for 24 h. Then, the cell monolayers were washed in PBS, cultured for an additional 24 h, and selected for 2 days with 1 μg/mL puromycin (Beyotime, China).

Coimmunoprecipitation. Coimmunoprecipitation was performed as previously described^29^. BMSCs were transfected with lentivirus encoding 3×Flag-FBLN2. The transfected cells were cultured for 36 to 48 h. For immunoprecipitation of 3×Flag-tagged Fibulin2 and Notch2, the cells were lysed in lysis buffer (Thermo Fisher, USA). The whole cell lysates were precleared with rProtein G Sepharose (Thermo Fisher, USA) and 2 µg of control IgG (Abcam, ab172730) at 4 °C for 1 h. The supernatants were collected and incubated at 4 °C for 4 h with either anti-3×Flag antibody (CST, 14793S), anti-Notch2 antibody (CST, 7532S), or control IgG (Abcam, ab172730). Then, the immune complexes were collected after incubation for 1 h at 4 °C with rProtein G Sepharose (Thermo Fisher, USA). After three washes in wash buffer, the immunoprecipitates were boiled in 2X loading buffer (Beijing Biomed Gene Technology Co.) for 10 min and subjected to immunoblotting.

### Protein modeling and molecular docking

Sequence search-based modeling of Fibulin2 was performed using the SWISS-MODEL database (swissmodel.expasy.org) based on the structure of Dll1 (PDB ID: 4xbm.2. A, 31.44% shared identity). The SWISS-MODEL-predicted structures were analyzed for the QMEAN Z score, which included the cumulative Z score of the Cβ, all atoms, solvation and torsion values. The Notch2 domain structure was downloaded from the RCSB PDB database (www1.rcsb.org). The initial docking between the Fibulin2 and Notch2 structures was performed with Z-Dock (3.0.2). Then, model screening was conducted using PDBePISA to identify all conformations (www.ebi.ac.uk/msd-srv/ssm/).

### Binding assay

Binding between Fibulin2 and Notch2 was determined using a real-time BLI assay with an Octet RED2 biosensor (ForteBio, USA). The entire experiment was performed at 30 °C in PBS with Tween (0.02% PBST) buffer with plate shaking at 1000 rpm. Protein A biosensors (ForteBio, USA) were first loaded with 200 nM Notch2 protein (R&D Systems, USA) for 600 s and then with PBST for another 600 s to obtain the baseline. Then, the biosensors were dipped into Fibulin2 for 1200 s to obtain the binding signal and finally dipped into PBST to obtain data for the dissociation curve. PBST buffer was used as a negative control.

### Real-time quantitative PCR analysis

Total RNA was extracted from cells using a High Pure RNA isolation kit (BioTeke, China) and reverse transcribed into cDNA with a PrimeScript RT reagent kit (TaKaRa, PR037A, Japan). Real-time RT–PCR was performed on a Bio-Rad CFX96 system. The following primer sets were used: Gapdh-F, GGAGCGAGATCCCTCCAAAAT, and Gapdh-R, GGCTGTTGTCATACTTCTCATGG; Fibulin2-F, AGTCAGCCACTGTCCACCATCC, and Fibulin2-R, CTCACTGTCCTCGGTCACTCTCC; Col1a1-F, GAGGGCCAAGACGAAGACATC, and Col1a1-R, CAGATCACGTCATCGCACAAC; Alp-F, ACTCTCCGAGATGGTGGTGGTG, and Alp-R, CGTGGTCAATTCTGCCTCCTTCC; Runx2-F, AGGCAGTTCCCAAGCATTTCATCC, and Runx2-R, GGCAGGTAGGTGTGGTAGTGAG; Notch2-F, CCTTCCACTGTGAGTGTCTGA, and Notch2-R, AGGTAGCATCATTCTGGCAG; Hey1-F, GTTCGGCTCTAGGTTCCATGT, and Hey1-R, CGTCGGCGCTTCTCAATTATTC; and Hes1-F, ACGTGCGAGGGCGTTAATAC, and Hes1-R, GGGGTAGGTCATGGCATTGA.

### Immunoblotting

Briefly, cells were gently lysed by incubation in ice-cold RIPA buffer (Beyotime, China) containing protease inhibitors (Beyotime, China) for 30 min followed by centrifugation at 12,000 × *g* for 15 min; then, the supernatant was collected. The blots were probed with rabbit anti-human β-actin (Multi Science; 1:5000), rabbit anti-human Fibulin2 (Abcam, ab234993; 1:2000), rabbit anti-human Notch2 (Abcam, ab245325; 1:2000), rabbit anti-human Hes1 (Abcam, ab108937; 1:1000), rabbit anti-human Col1a1 (Abcam, ab260043; 1:1000), and rabbit anti-human Runx2 antibodies (Abcam, ab236639; 1:1000). The secondary antibody was peroxidase-conjugated goat anti-rabbit Ig (Abcam, ab205718; 1:5000). Protein ladder(10-180KD) was provided from Biosynthesis Biotechnology Inc. (Beijing, China). Densitometry was performed with ImageJ software.

### Transplantation of BMSCs

In total, 25 nude mice (6–8 weeks old, weighing 18–22 g) were assigned to 5 groups: the control, shFibulin2, scramble, Fibulin2, and vehicle groups. A unilateral 0.5-mm diameter full-thickness circular defect was generated in the middle of the right femur. This critical-size defect was filled with gelatin sponge. The control group was inoculated with 20 µl of osteogenic cell-free medium, while the other groups were inoculated with 20 µl of a BMSC suspension. Approximately 1 × 10^6^ BMSCs transfected with shFibulin2, Fibulin2, scramble or vehicle lentivirus were suspended in 20 μL of osteogenic medium before application to the defect. After 14 days, the femurs were collected, and intravital imaging, micro-CT, and histological experiments were performed.

### CLP procedure

CLP surgery was performed as reported previously^30^. In summary, mice were anesthetized. The cecum was exteriorized through a 0.8-cm midline abdominal incision and ligated distal to the ileocecal junction. Approximately 50% of the cecum was ligated. The distal cecum was punctured bilaterally with a 23-gauge needle. A small amount of luminal content was pushed through both puncture sites to ensure patency. The cecum was returned to the abdominal cavity, and the incisions were closed. The sham group mice underwent identical procedures without ligation or puncture of the cecum. All animals received subcutaneous fluid resuscitation with 1.0 mL of saline immediately after surgery. The femurs were harvested 28 days after surgery and used for micro-CT and histological experiments.

### Intravital imaging

Experimental animals were anesthetized using 4% chloral hydrate (100 µL/10 g) and then euthanized, and the right femur was obtained. Following careful dissection of the skin and muscle covering the bone, the femur was placed on the stage of an imager (Fusion FX6 Edge). The control group served as the negative control group, and femurs from the other groups were selected for further imaging. All images were analyzed using the Fusion FX6 Edge software platform. Then, femurs harvested 28 days after surgery were used in other experiments.

### Three-dimensional microcomputed tomography (micro-CT) analyses

Mouse femurs were dissected and scanned by micro-CT (vivaCT40, SCANCO Medical AG, Switzerland) at 70 kV and 114 μA. The scans were reconstructed by micro-CT Evolution Program version 5.0 software. For analysis of the healing of the 0.5-mm drill hole defect in the femur, images were reoriented using the same filtering and segmentation values used to obtain the 3D images. The analyzed parameters included BS, BV, BV/TV, Tb. Th, and Tb. N.

### Construction of *Fibulin2*−/− mice

*Fibulin2*−/− mice were constructed by Cyagen Biosciences Inc. (Suzhou, China) using the CRISPR/Cas9 system. The mouse *Fibulin2* gene (Gene ID: 14115; Ensembl: ENSMUSG00000064 080) is located on chromosome 6 and contains 18 exons. For generation of *Fibulin2-*KO mice, exon 2 was deleted in WT C57BL/6 mice. The *Fibulin2-*KO mice were genotyped by PCR and subsequent DNA sequencing analysis. Homozygous mice were generated by crossing heterozygous mice.

### Whole skeleton staining

Whole neonatal mouse skeletons were subjected to ARS/Alcian blue staining according to a published method^31^. In summary, neonatal mice were sacrificed on Days 0, 7 or 14; fixed in 10% neutral buffered formalin; rinsed with double-distilled H_2_O; and postfixed with 70% ethanol. Then, we removed the skin and internal organs and stained the cartilage with 0.2% Alcian blue 8GX (Sigma, Germany) in glacial acetic acid. The mice were treated with 1.0% trypsin in 30% saturated sodium borate before mineralized bone was immersed in ARS (Sigma, Germany) in 0.5% KOH and then treated with a graded series of a KOH-glycerol solution. Finally, we collected images with a high-resolution scanner.

### Statistical analyses

Statistical analyses were performed using ImageJ, SPSS 25 and GraphPad Prism 8 software. Cell-based experiments were performed at least twice. Animals were randomly assigned to different groups, and unless otherwise stated, at least 3 mice were included in each group. Data are presented as the mean ± SD. A two-tailed *t* test was used to compare differences between two groups, and ordinary one-way ANOVA was used to compare differences among more than two groups. Statistically significant differences are indicated by *p* < 0.05.

## Results

### Fibulin2 is highly expressed in infected bone tissues of patients with osteomyelitis

**Fig. 1:**
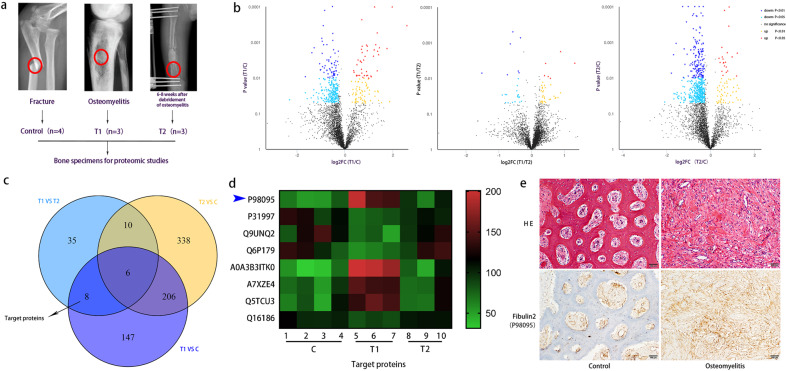
Fibulin2 is highly expressed in infected bone tissues of patients with osteomyelitis. **a** Schematic diagram showing the bone tissue subjected to proteomic analyses. Bone samples were collected from the areas indicated by the red rings. **b** Volcano plots presenting the differentially expressed proteins identified through comparisons between two groups. **c** Venn diagram showing the significant differences in 8 target proteins in the osteomyelitis and normal control samples and in the osteomyelitis samples before and 6–8 weeks after debridement. **d** Heatmap showing 8 target proteins. P98095 (blue arrow) was selected for further research. **e** HE and Fibulin2 (P98095) staining of normal and infected bone. HE staining showed obvious inflammatory cell infiltration and bone destruction in the sections with osteomyelitis. Moreover, locally enhanced Fibulin2 expression was captured in approximately the same infected areas.

To identify molecules significantly involved in bone infection, we previously performed a standard proteomic analysis of bone tissues from patients with osteomyelitis, fracture or femoral head necrosis and from patients who had recovered from osteomyelitis (6–8 weeks after debridement) (Fig. 1a). The proteomic data were then qualitatively, quantitatively and functionally analyzed. Differentially expressed proteins identified by comparison of any two groups are presented in a volcano plot (Fig. 1b). Then, the three groups of identified proteins were compared, and we selected target proteins with elevated expression in the osteomyelitis group compared to the control group. From this list, we identified those proteins whose levels decreased after the bone infection cleared, and we found no significant difference in the expression of these proteins in the control volunteers and the patients who had recovered from osteomyelitis (Fig. 1c). The target proteins, namely, P31997, P98095, Q9UNQ2, Q6P179, A0A3B3ITK0, A7XZE4, Q5TCU3, and Q16186, are presented in a heatmap (Fig. 1d). Literature consultation led to the identification of Fibulin2 (P98095), a secreted extracellular matrix (ECM) glycoprotein. Then, hematoxylin and eosin (HE) staining and immunohistochemistry were performed to estimate Fibulin2 expression in paraffin‐embedded sections of infected bone and surrounding uninfected osseous tissue. As shown in the HE images, inflammatory cells infiltrated the infected bone section, which showed tissue destruction. In addition, locally enhanced Fibulin2 expression was observed in approximately the same infected areas (Fig. 1e). These results suggested that the expression of Fibulin2 was upregulated in infected bone tissue.

### Fibulin2 KO accelerates bone formation during early skeletal development and inhibits inflammatory bone resorption after cecal ligation and puncture (CLP) surgery in mice

**Fig. 2:**
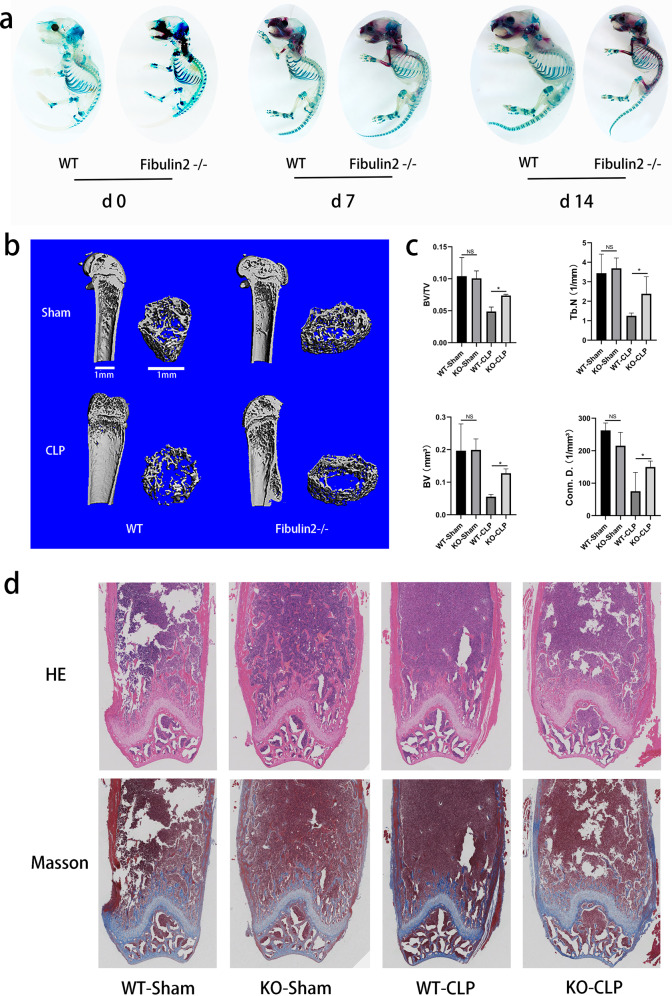
Fibulin2 knockout accelerates bone formation during early skeletal development and inhibits inflammatory bone resorption after cecal ligation and puncture surgery in mice. **a** Fibulin2 knockout (KO) neonatal mice and wild-type (WT) mice from the same litter were used to stain whole skeletal tissue with alizarin red/Alcian blue, and the staining intensity was assessed after 0, 7 or 14 days. Mineralized bone was stained red, and cartilage was stained blue. **b** Trabeculae in the area within 1 cm of the proximal end of the growth plate in femurs. Trabecular number was determined by microcomputed tomography (micro-CT). **c** Quantification of the micro-CT results. Bone volume (BV), bone volume/tissue volume (BV/TV), trabecular bone number (Tb. N), and connectivity density (Conn. D.). (NS not significant; *P < 0.05, *n* ≥ 3). **d** HE-stained and Masson’s trichrome-stained femurs. (CLP cecal ligation and puncture). All femurs were harvested 28 days after surgery.

To explore the potential regulatory effect of Fibulin2 on skeletal formation, we generated *Fibulin2−/*− mice. We compared mineralized bone formation in *Fibulin2−/*− neonatal mice and wild-type (WT) mice from the same litters to evaluate skeletal development. Whole skeleton staining on postnatal Days 0, 7 and 14 confirmed that skeletal mineralization was notably accelerated in the *Fibulin2−/*− neonatal mice compared with the WT mice (Fig. 2a). The forelimbs, skull bone and rib cage appeared to show greater mineralization in the KO mice at Days 0, 7 and 14 (Supplementary Fig. 1a, c, d). The mineralization rate of the hindlimbs and lower vertebral column was obviously higher on Day 14 in the *Fibulin2−/−* neonatal mice than in the WT mice, but the Fibulin2-KO mice did not exhibit hypermineralization on Day 0 or 7 (Supplementary Fig. 1b, e).

Infection models were established by subjecting 6- to 8-week-old adult mice to CLP surgery. Femurs were harvested 28 days post-surgery, and bone resorption differences in the *Fibulin2−/*− mice and the WT mice were determined. Microcomputed tomography (micro-CT) imaging indicated no significant difference in the volume ratio of mineralized bone to total bone (BV/TV), trabecular bone number (Tb. N), bone volume (BV), or connectivity density (Conn. D) between the adult *Fibulin2−/*− and WT mouse sham groups. However, after CLP surgery, the BV/TV, Tb. N, BV, and Conn. D were higher in the *Fibulin2-/-* mice than in the WT mice (Fig. 2b, c). The HE and Masson’s trichrome staining results revealed many more trabeculae in the sham groups. On Day 28 post-surgery, there were fewer trabeculae in the femurs of both *Fibulin2−/*− and WT mice, but the trabecular count was lower in the WT mice than in the *Fibulin2-/-* mice (Fig. 2d). The above results suggested that Fibulin2 plays a key regulatory role in early skeletal development and inflammatory bone resorption induced by infection.

### Fibulin2 expression is upregulated in BMSCs upon lipopolysaccharide (LPS) or peptidoglycan (PGN) treatment

BMSCs were isolated from human bone marrow and identified by surface marker expression and multidifferentiation potential. The extracted BMSCs met the International Society for Cellular Therapy criteria^32^; that is, they showed adherence plasticity; expressed CD105, CD90 and CD73; and did not express CD45, CD34, CD11b, CD19 or HLA-DR. Moreover, they differentiated into adipocytes, chondrocytes and osteoblasts after induction (Supplementary Fig. 2).

**Fig. 3:**
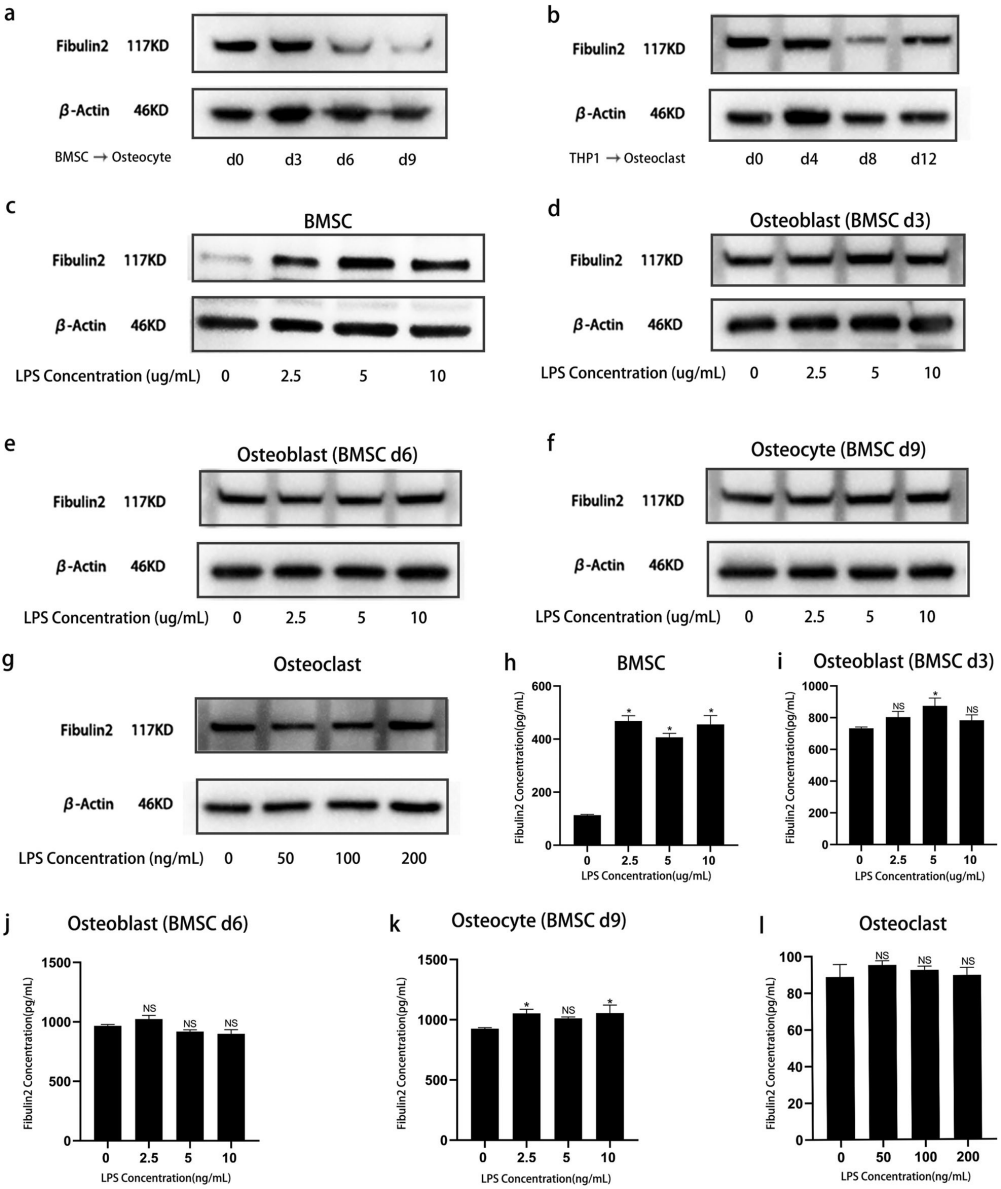
Fibulin2 expression in different cells deep within the bone. **a** The differential expression of Fibulin2 in the BMSCs differentiated into osteocytes was detected by western blotting (WB). **b** The differential expression of Fibulin2 in the THP-1 cells differentiated into osteoclasts was detected by WB. The expression of Fibulin2 in BMSCs (**c**), osteoblasts (**d** and **e**), osteocytes (**f**), and osteoclasts (**g**) after stimulation with lipopolysaccharide (LPS) for 24 h was detected by WB. The expression of Fibulin2 in BMSCs (**h**), osteoblasts (**i** and **j**), osteocytes (**k**), and osteoclasts (**l**) after stimulation with LPS for 24 h was detected by ELISAs. Osteoblasts were derived from BMSCs after induction of osteogenic differentiation for 3 or 6 days; osteocytes were derived from BMSCs after osteogenic differentiation for 9 days; and osteoclasts were derived from THP-1 cells after osteoclast differentiation for 12 days.

To investigate whether Fibulin2 is related to the differentiation of osteocytes and osteoclasts, we measured Fibulin2 expression during the differentiation process by WB. The results showed that Fibulin2 expression was downregulated when BMSCs differentiated into osteocytes and when THP-1 cells differentiated into osteoclasts (Fig. 3a, b). As BMSCs, osteoblasts, osteocytes and osteoclasts are the major cellular components of human bone^33^, we stimulated BMSCs with LPS for 24 h and then detected Fibulin2 expression. The culture supernatants and cell lysates were separately analyzed to determine the levels of secreted and intracellular Fibulin2. The WB results showed that Fibulin2 expression was upregulated in LPS-stimulated BMSCs (Fig. 3c). However, LPS treatment did not significantly influence Fibulin2 expression in osteoblasts, osteocytes or osteoclasts (Fig. 3d–g). Moreover, LPS significantly increased Fibulin2 secretion by BMSCs (Fig. 3h) but not by osteoblasts (Fig. 3i, j), osteocytes (Fig. 3k) or osteoclasts (Fig. 3l), as determined by ELISAs. Considering these results, we focused our study on BMSCs.

First, we stimulated BMSCs with PGN and found that this important bacterial component upregulated Fibulin2 expression (Supplementary Fig. 3a–c). Then, we stimulated the common osteoblast progenitor HOS and 9901 cell lines with LPS and found that Fibulin2 expression was upregulated in both HOS cells (Supplementary Fig. 3d–f) and 9901 cells (Supplementary Fig. 3g–i). These results suggested that infection-induced Fibulin2 originates in BMSCs or osteoblast progenitor cells in bone tissue.

### Fibulin2 negatively regulates the osteogenic differentiation of BMSCs in vitro

**Fig. 4:**
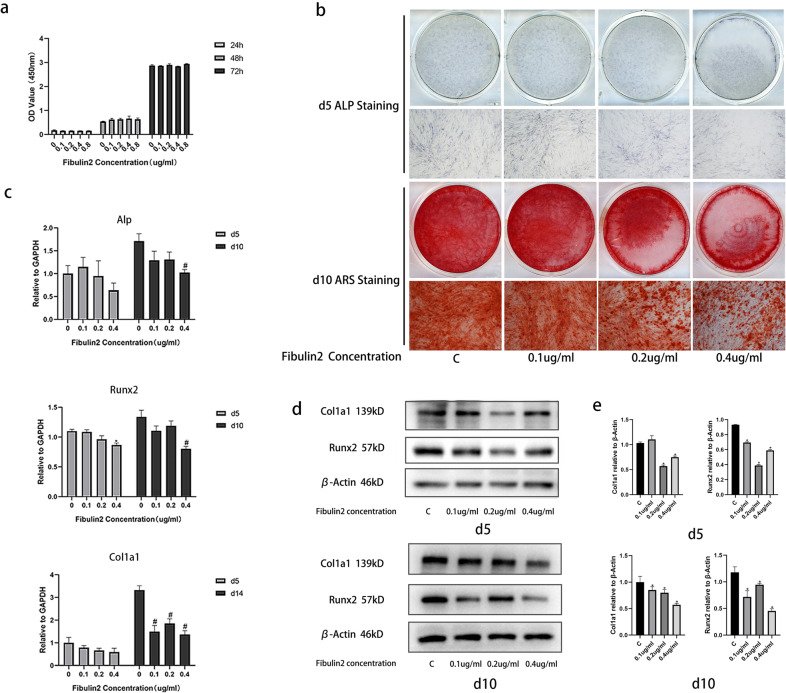
Fibulin2 negatively regulates the osteogenic differentiation of BMSCs in vitro. **a** Cell Counting Kit-8 (CCK-8) data revealed no differences in the optical density (OD) value of the BMSCs treated with different concentrations of Fibulin2 at 24, 48, or 72 h. **b** Alkaline phosphatase (ALP) staining on Day 5 and alizarin red staining (ARS) on Day 10 were performed after the BMSCs were stimulated with different concentrations of Fibulin2 to induce osteogenic differentiation. Reduced Alp activity was found at 5 days, and fewer calcium nodules were identified 10 days after stimulation with Fibulin2. **c** The expression of Alp, Runx2 and Col1a1 in BMSCs was detected by RT-qPCR. (*the Fibulin2 group vs. the control group, *P* < 0.05 on Day 5; #the Fibulin2 group vs. the control group, *P* < 0.05 on Day 10; *n* = 3). **d** The expression of Col1a1 and Runx2 in BMSCs as detected by western blotting (WB). **e** Quantification of WB data (*the Fibulin2 group vs. the control group, *P* < 0.05; *n* = 3).

We first explored the proliferative or cytotoxic effects of Fibulin2 on BMSCs. Cell Counting Kit-8 (CCK-8) assays confirmed that Fibulin2 did not promote cell proliferation or cause toxicity; specifically, Fibulin2 treatment (0.1–0.8 µg/mL) did not change the number of BMSCs after 24, 48 or 72 h (Fig. 4a). Then, we evaluated the effects of Fibulin2 on osteogenesis in vitro by measuring the osteogenic differentiation of Fibulin2-treated human BMSCs based on active alkaline phosphatase (Alp) staining on Day 5 and alizarin red staining (ARS) on Day 10. Compared to the control, Fibulin2 decreased both Alp staining on Day 5 and ARS on Day 10 (Fig. 4b). Furthermore, the expression levels of osteogenic differentiation markers (Alp, Runx2, and Col1a1^34^) were determined by RT–qPCR and WB. After 5 or 10 days of Fibulin2 treatment, the mRNA levels of Alp, Runx2, and Col1a1 were decreased (Fig. 4c). The protein levels of Runx2 and Col1a1 were similarly reduced on Days 5 and 10 (Fig. 4d, e). These results suggested that Fibulin2 negatively regulates the osteogenic differentiation of BMSCs.

### Fibulin2 suppresses BMSC osteogenic differentiation by directly binding to Notch2 and inactivating Notch2 signaling pathways

**Fig. 5:**
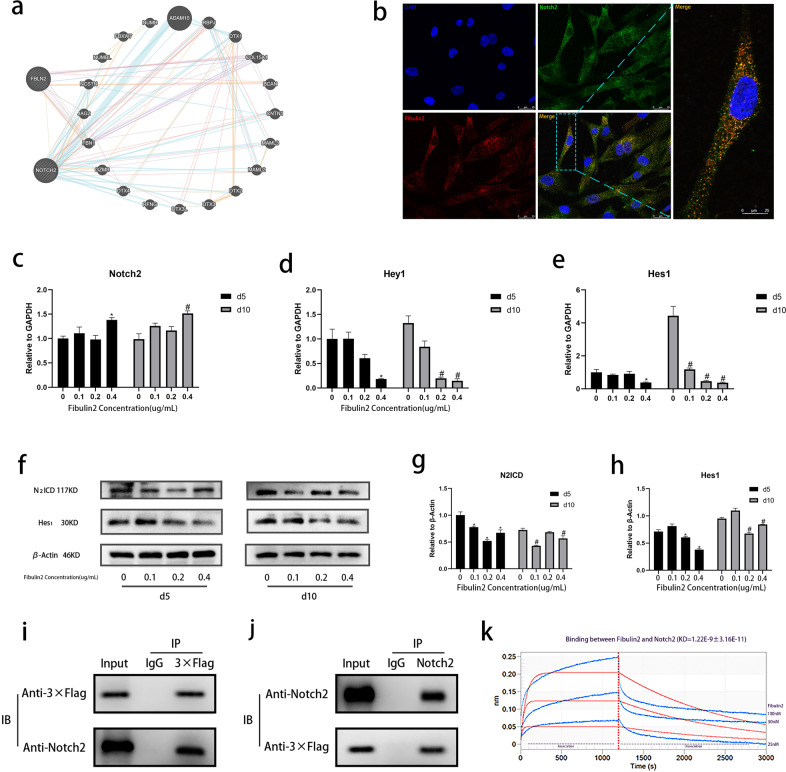
Fibulin2 interacts with Notch2 and inactivates the Notch2 signaling pathway. **a** A bioinformatics analysis-based prediction suggested that Fibulin2 may interact with Notch2. **b** Double-labeling immunofluorescence staining revealed that Fibulin2 colocalized with Notch2 in BMSCs. The area with a dotted line is magnified and shown on the right. **c**–**e** Expression of Notch2 pathway genes. (*the Fibulin2 group vs. the control group, *P* < 0.05 on Day 5; #the Fibulin2 group vs. the control group, P < 0.05 on Day 10; n = 3). **f** WB was performed to detect N2ICD and Hes1 expression levels. **g, h** Quantification by WB. (*the Fibulin2 group vs. the control group, *P* < 0.05 on Day 5; #the Fibulin2 group vs. the control group, *P* < 0.05 on Day 10; *n* = 3). **i, j** Coimmunoprecipitation of Fibulin2 and Notch2. BMSCs were transfected with lentivirus expressing 3×Flag-tagged Fibulin2. **i** Cell lysates were immunoprecipitated with anti-3×Flag antibody or control IgG. The immunoprecipitates were immunoblotted with anti-Notch2 antibody. **j** Cell lysates were immunoprecipitated with anti-Notch2 antibody or control IgG. The immunoprecipitates were immunoblotted with anti-3×Flag antibody. **k** BLI-based biosensor technology was applied to demonstrate that Fibulin2 (Ala28–Leu1184) bound to Notch2 (Leu26–Gln530). The blue curves represent the true association and dissociation signals. The red curves show the curve fitting results. KD = 1.22 × 10^−9^ ± 3.16 × 10^−11^.

We next investigated the mechanisms by which Fibulin2 negatively regulates osteogenic differentiation. First, we performed a bioinformatics analysis to predict the receptors of Fibulin2 and the corresponding signaling pathways. Fortunately, we successfully identified a potentially relevant relationship between Fibulin2 and Notch2 (Fig. 5a). In this study, double-labeling immunofluorescence staining revealed the colocalization of Fibulin2 and Notch2 in BMSCs (Fig. 5b).

The evolutionarily conserved Notch signaling pathway determines cell fate in diverse tissues^35^. Notch is activated through cleavage by γ-secretase, and the intracellular domain translocates to the nucleus, where it modulates the transcription of target genes, such as hairy/enhancer of split (Hes) and Hes-related protein (Hey) gene family members^36,37^. Notch2 is a member of the Notch family, and Notch2 intracellular domain (N2ICD) protein expression indicates activation of the Notch2 pathway. To determine the effect of Fibulin2 on Notch2 expression and signaling, we detected the expression of factors downstream of the Notch2 pathway in BMSCs treated with Fibulin2. RT-qPCR assays showed that Fibulin2 did not downregulate Notch2 mRNA expression (Fig. 5c) but did downregulate the mRNA expression of downstream molecules, including Hes1 and Hey1 (Fig. 5d, e). In addition, WB revealed that N2ICD and Hes1 protein expression was decreased (Fig. 5f–h). Taken together, these results suggested that Fibulin2 expression in BMSCs may inactivate the Notch2 signaling pathway.

To determine the interaction of Fibulin2 and Notch2, we transfected BMSCs with lentivirus expressing 3×Flag-tagged Fibulin2. Subsequently, cell lysates were immunoprecipitated with either anti-3×Flag antibody, anti-Notch2 antibody or control IgG, and then, Notch2 and 3×Flag-tagged Fibulin2 were detected using an anti-Notch2 antibody and anti-3×Flag antibody, respectively. The results showed that Fibulin2 interacts with Notch2 in BMSCs (Fig. 5i, j). In addition, using biolayer interferometry (BLI)-based biosensor technology, we investigated whether Fibulin2 shows any binding affinity for Notch2. In a gradient experiment, proportional dilutions of Fibulin2 (25, 50, and 100 nM) were allowed to associate with Notch2. Fibulin2 (Ala28–Leu1184) displayed high binding affinity for Notch2 (Leu26–Gln530) with KD = 1.22 × 10^−9^ ± 3.16 × 10^−11^ (Fig. 5k).

These results prompted us to further characterize the mode of the interaction between Fibulin2 and Notch2. Homology structure modeling is a common way to rebuild target protein structures. A previous study clarified that 3D protein structures are similar when the shared sequence identity is higher than 25%^38^. To obtain a proper template, we searched the SWISS-MODEL database using the Fibulin2 sequence. With Dll1 (4xbm.2. A) as the template, the final Fibulin2 model was obtained through homology modeling and structural optimization; the sequence identity between Fibulin2 and the homologous template was 31.44% (Supplementary Fig. 4a). The protein spatial structure was found to adopt an L-shape (Supplementary Fig. 4b). The structure quality assessment revealed that the QMEAN, Cβ, All Atom, solvation, and torsion values were −4.85, −3.06, −4.2, −3.12, and −3.42, respectively (Supplementary Fig. 4c). The Notch2 domain structure was then downloaded from the RCSB PDB. The initial docking between the Fibulin2 and Notch2 structures was performed using Z-Dock (3.0.2), which offers a rigid body-based docking protocol based on the fast Fourier transform (FFT) algorithm to perform a 3D search of all possible binding modes of two protein structures in both translational and rotational space^39^. By docking Fibulin2 with the Notch2 domain via Z-Dock, 10 ligand–receptor complex models were obtained. Then, the models were screened using PDBePISA to identify all possible conformations. The best docked complex is presented in Supplementary Fig. 4d. As shown in the graph, the docking complex is shaped as a J or a hook. In addition, the molecular docking interface is presented in Supplementary Fig. 4e. Taken together, these results suggested that Fibulin2 suppresses the osteogenic differentiation of BMSCs by directly binding to Notch2 and inactivating the Notch2 signaling pathway.

### Overexpression of Fibulin2 blocks BMSC osteogenesis by inhibiting Notch2 pathway activation

To verify the negative regulatory effect of endogenous Fibulin2 on BMSC osteogenesis, we transfected BMSCs with control and Fibulin2-expressing lentiviral (Lv) vectors. RT–qPCR and WB analyses showed that Fibulin2 levels were highly increased in the BMSCs transfected with the Lv-Fibulin2 vector compared to those transfected with the control vector (Supplementary Fig. 5a). We next examined the osteogenic ability of BMSCs infected with Lv-Fibulin2 or control. As shown by the Alp activity assay on Day 5 and ARS on Day 10, Fibulin2 overexpression inhibited the osteogenesis of BMSCs (Supplementary Fig. 5b). To understand the intrinsic mechanism by which Fibulin2 overexpression contributes to slowed bone formation, we detected the expression of relevant osteogenesis-related markers and Notch2 pathway components. The RT–qPCR results showed that Alp, Runx2, Col1a1, Hey1 and Hes1 transcript levels were decreased in the Fibulin2 overexpression group compared to the control group (Supplementary Fig. 5c). WB showed that the protein levels of Runx2, Col1a1, N2ICD and Hes1 were similarly decreased (Supplementary Fig. 5d, e).

### Knocking down Fibulin2 expression enhances BMSC osteogenesis, and this excessive osteogenesis is abolished by DAPT treatment

**Fig. 6:**
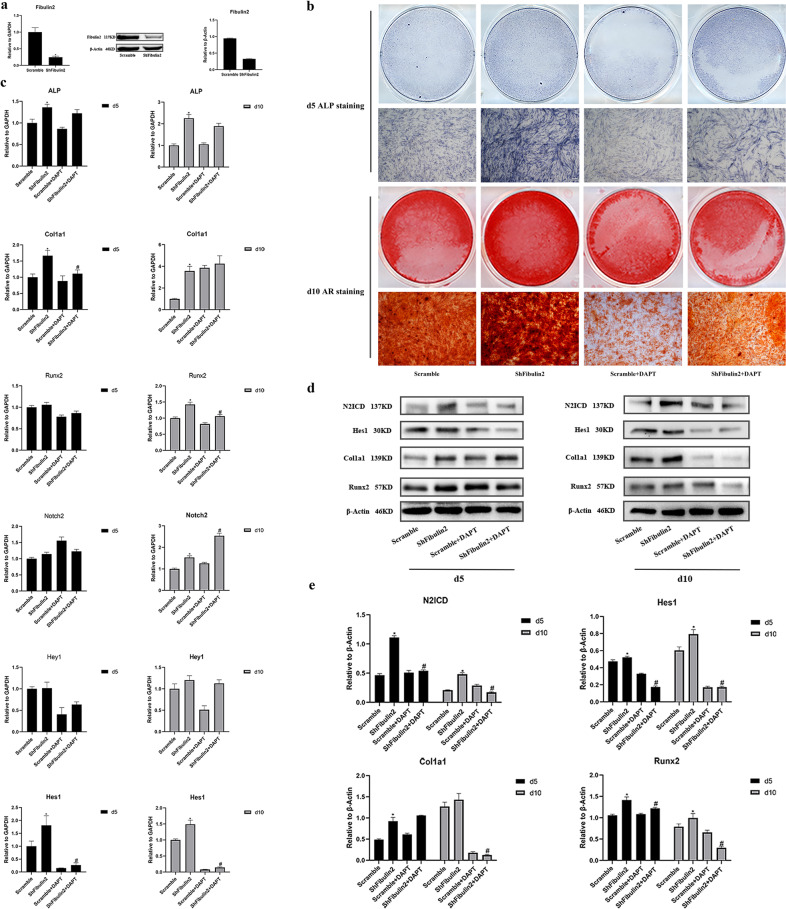
Fibulin2 knockdown enhances BMSC osteogenesis, and excessive osteogenesis is abolished by DAPT. **a** Effective knockdown of Fibulin2 in BMSCs was confirmed by RT–qPCR and western blotting (WB). **b** Alkaline phosphatase (ALP) staining on Day 5 and alizarin red staining (ARS) on Day 10 were performed after the BMSCs were transfected with different lentiviruses during osteogenic differentiation. Alp staining and ARS were more intense on Days 5 and 10, respectively, in the shFibulin2 group than in the scramble group, while DAPT reversed the increased osteogenic differentiation, as evidenced by less intense staining in the scramble + DAPT and shFibulin2 + DAPT groups. **c** The mRNA expression of osteogenic differentiation-related genes (ALP, Runx2, and Col1a1) and Notch2 pathway-related genes was upregulated in the shFibulin2 group compared to the control group and was decreased after DAPT treatment. (*the shFibulin2 group vs. the control group, *P* < 0.05; #the shFibulin2 + DAPT group vs. the shFibulin2 group, *P* < 0.05; *n* = 3). **d** WB indicated that BMSCs expressing shFibulin2 had higher protein expression of Hes1, N2ICD, Runx2 and Col1a1; however, the expression of these proteins was decreased in the DAPT treatment group. **e** Quantification of the WB data. (*the shFibulin2 group vs. the control group, *P* < 0.05; #the shFibulin2 + DAPT group vs. the shFibulin2 group, *P* < 0.05; *n* = 3). Scramble: BMSCs transfected with control lentivirus; shFibulin2: BMSCs transfected with lentivirus harboring shRNA targeting Fibulin2.

We employed Lv vectors expressing short hairpin RNA (shRNA) to specifically knockdown Fibulin2 in cultured BMSCs. Effective Fibulin2 knockdown in BMSCs was confirmed by RT–qPCR and WB. Transfection of BMSCs with shFibulin2 resulted in significantly lower expression of Fibulin2 than transfection with scramble shRNA (Fig. 6a). To provide further evidence for the roles of Fibulin2 and Notch2 in BMSC osteogenesis, we treated transfected BMSCs with the Notch2 signaling pathway inhibitor DAPT. The enhanced osteogenic differentiation of BMSCs with downregulated Fibulin2 expression was reversed by treatment with 10 μM DAPT. Alp staining and ARS of the shFibulin2-BMSCs appeared more intense on Days 5 and 10, respectively, after osteogenic induction, and DAPT reversed this enhanced osteogenic differentiation, as indicated by decreased Alp activity and ARS in the scramble + DAPT group compared with the shFibulin2 + DAPT group (Fig. 6b). Consistent with the staining results, the mRNA expression of osteogenic differentiation-related genes (Alp, Runx2, and Col1a1) was upregulated in the shFibulin2 group compared to the scramble group but was downregulated after DAPT treatment (Fig. 6c). To verify the gene expression data, we performed WB. The shFibulin2-BMSCs had higher protein expression of Hes1, N2ICD, Runx2 and Col1a1; however, the expression of these proteins was downregulated in the DAPT treatment group (Fig. 6d, e).

### Fibulin2 is a negative regulator of BMSC-mediated bone repair

**Fig. 7:**
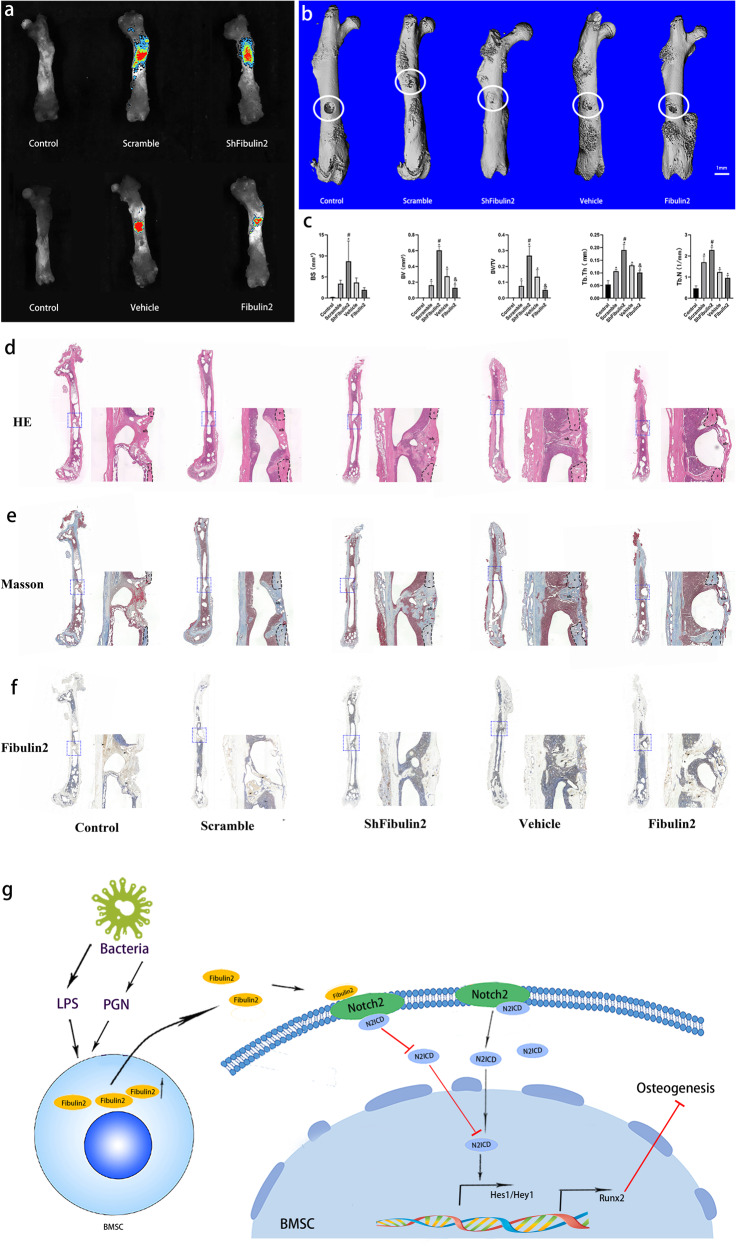
Fibulin2 is a negative regulator of BMSC-mediated bone repair. **a** Fluorescence tracking of BMSCs transfected with lentivirus using bioluminescence imaging. The BMSCs survived 14 days after transplantation into holes drilled into the femurs of nude mice. **b** Drill hole closure was determined with microcomputed tomography (micro-CT) 14 days after BMSC transplantation. The white circles indicate the bone area with drilled holes. **c** Quantification of the micro-CT results. Bone surface (BS), bone volume (BV), bone volume/tissue volume (BV/TV), trabecular bone thickness (Tb. Th), and trabecular bone number (Tb. N) were increased in the shFibulin2 group and decreased in the Fibulin2 group. (*The vehicle or scramble group vs. the control group, *P* < 0.05; # the shFibulin2 group vs. the scramble group, *P* < 0.05; &the Fibulin2 group vs. the vehicle group, *P* < 0.05; *n* ≥ 3). **d**–**f** Histology and immunohistochemistry analyses. Hematoxylin and eosin (HE) staining (**d**) and Masson’s trichrome staining (**e**) showed greater new bone formation in the shFibulin2 group than in the scramble group and less new bone formation in the vehicle group than in the Fibulin2 group. Fibulin2 staining (**f**) indicated that Fibulin2 was expressed at higher levels in the areas with bone defects but at lower levels in areas with new bone. The “c” circled with a black dotted line represents an area of the mouse cortex, “nb” indicates new bone, black arrows highlight the accumulation of Fibulin2, and the images on the right are 10-fold magnifications of the blue boxed regions. Control group: osteogenic differentiation medium without cells was transplanted into bone with drilled holes; scramble and vehicle groups: BMSCs transfected with different lentiviruses (scramble, vehicle) were transplanted into bone with drilled holes in osteogenic differentiation medium; shFibulin2 group: BMSCs transfected with lentivirus expressing shRNA targeting Fibulin2; and Fibulin2 group, BMSCs transfected with lentivirus to upregulate Fibulin2 expression. **g** Schematic diagram of the involvement of Fibulin2 in bone infection. In infected bone, pathogenic bacteria release lipopolysaccharide (LPS) or peptidoglycan (PGN), which results in the enhanced secretion of Fibulin2 by BMSCs. Then, Fibulin2 binds to the membrane receptor Notch2. The association of Fibulin2 with Notch2 inhibits the activation of the Notch2 pathway, reducing N2ICD release and thereby decreasing the transcription of Hes1, Hey1 and Runx2. The final result is the inhibition of BMSC osteogenesis.

To determine the role of Fibulin2 in skeletal recovery after injury, we assessed bone regeneration in nude mice with artificial bone defects that underwent BMSC transplantation. Bioluminescence images demonstrated that BMSCs survived 14 days after transplantation (Fig. 7a). Then, using micro-CT, we measured the degree to which a drill hole was closed. The results showed that 14 days after injury, the volume of newly formed bone tissue in the drill hole was smaller in the mice transplanted with the BMSCs overexpressing Fibulin2 than in those in the vehicle group. Greater new bone growth in the drill hole was observed in the mice transplanted with the shFibulin2-expressing BMSCs than in those transplanted with the scramble-expressing BMSCs (Fig. 7b). Quantitative analyses of micro-CT images revealed that the vehicle and shFibulin2 groups exhibited greater osteogenic ability than the Fibulin2 and scramble groups, as indicated by the marked increases in bone surface (BS), BV, BV/TV, trabecular bone thickness (Tb. Th), and Tb. N (Fig. 7c). Then, histological and immunohistochemical analyses of mouse femurs were performed. HE and Masson’s trichrome staining showed greater new bone formation in the shFibulin2 group than in the scramble group and in the vehicle group than in the Fibulin2 group (Fig. 7d, e). Moreover, staining indicated that Fibulin2 was expressed at higher levels in the bone defect area and at lower levels in the new bone area (Fig. 7f). Taken together, these findings suggest that Fibulin2 plays a negative regulatory role in BMSC-mediated bone repair.

## Discussion

Bone homeostasis is a dynamic state maintained by the relative balance between bone formation and bone absorption. This equilibrium is tightly regulated by osteoclasts and osteoblasts, which originate from BMSCs^40–42^. In our previous studies, we demonstrated that components of bacteria, such as staphylococcal protein A and LPS, activate osteoclasts, thereby subtly disrupting bone homeostasis and leading to osteolysis and skeletal necrosis^13,43,44^. To gain a deeper understanding of the mechanism by which bone infection contributes to delayed union or nonunion, we performed proteomic research with collected bone samples to identify molecules that effectively disturb osteogenesis. Proteomic analyses revealed significantly higher levels of Fibulin2 in the bone tissue of osteomyelitis patients. In addition, secreted and intracellular Fibulin2 protein levels were abundant in BMSCs after LPS or PGN stimulation. The elevated Fibulin2 levels impeded the osteogenic differentiation of BMSCs, which was mediated by the attenuated activation of Notch2 (Fig. 7g).

In this study, our observation of increased Fibulin2 expression in infected bone tissue of patients led us to speculate that Fibulin2 may play a role in osteomyelitis. As important precursors of osteoblasts, BMSCs were analyzed to identify the mechanism by which Fibulin2 contributes to osteomyelitis. An important compound in gram-negative cells, LPS undoubtedly induces inflammation, potentially leading to inflammatory bone loss^43,45^. Hence, we stimulated BMSCs with different doses of LPS in vitro and found increased secreted and cytoplasmic Fibulin2 protein levels.

Next, a set of exploratory experiments was conducted to characterize the specific role of Fibulin2 in the progression of bone infection. We first treated BMSCs with different concentrations of recombinant Fibulin2 (rhFibulin2) protein. However, this treatment did not increase BMSC proliferation, induce cytotoxicity, or affect chemotaxis (data not shown). However, we found that Fibulin2 slowed BMSC osteogenesis. Numerous ECM proteins, including Noggin, connective tissue growth factor (CCN), fibrillin2, and fibulin1, have been previously reported to regulate osteogenesis^46–49^. Moreover, many studies have indicated that Fibulin2 is an ECM protein that can interact with numerous other ECM proteins^50^. However, little is known about the Fibulin2-induced regulation of bone remodeling. In this study, we investigated the precise role of Fibulin2 in the osteogenic differentiation of BMSCs. Our results showed that rhFibulin2 inhibited osteogenesis by decreasing Alp activity, calcium deposition, and the expression of osteogenic-related factors, such as Alp, Col1a1, and Runx2.

The evolutionarily conserved Notch signaling pathway plays important roles in cell fate determination^51^. Notch activation leads to a succession of cleavage events, including the generation of N2ICD. When Notch2 is cleaved, N2ICD is released and thought to translocate to the nucleus, where it stimulates the expression of downstream genes such as Hes1 and Hey1^25^. Importantly, Hes1 is thought to promote the transcription of Runx2, which is indispensable for osteogenesis^52^. Therefore, we evaluated N2ICD, Hes1 and Hey1 expression in BMSCs and found that Fibulin2 treatment successfully blocked the activation of the Notch2 pathway. Previous studies on the role of Notch2 in osteogenesis and bone formation have reported controversial results. Specifically, some researchers have reported that Notch2 activation is essential for osteogenic differentiation and identified Notch2 as an important effector in the osteoblastic differentiation of human periodontal ligament cells^53^. Moreover, Vollesren et al.^54^ implicated Notch2 mutants in Hajdu–Cheney syndrome, which is characterized by progressive osteoporosis. These findings offer evidentiary support for our results that Fibulin2 significantly repressed BMSC osteogenesis by blocking Notch2 pathway activation. However, other scientists have reported that Notch2 inactivation contributes to osteogenesis. Specifically, Yorgan et al.^55^ noted that Notch2 inactivation augmented bone mass in the appendicular skeleton. These contradictory outcomes likely stem from species differences. For example, Fengchang et al.^56^ demonstrated that Notch2 activation inhibited mouse MSC osteogenesis but enhanced human MSC osteogenic differentiation.

In the next step of the present study, we sought to verify the relationships among Fibulin2, Notch2 and BMSC osteogenesis. To accomplish this, we upregulated and downregulated Fibulin2 expression in BMSCs via Lv-Fibulin2 and Lv-shFibulin2 transfection and monitored the effects on Notch2 signaling pathway activation and osteogenesis in vitro. The results showed that Fibulin2 significantly inhibited the osteogenic differentiation of BMSCs by blocking Notch2 activation. Moreover, knocking down Fibulin2 expression increased bone formation via Notch2 activation. In addition, we treated Fibulin2 knockdown BMSCs with DAPT, a specific Notch inhibitor. The enhanced bone formation and Notch2 activation were abolished by DAPT treatment. Furthermore, in vivo experiments showed that Fibulin2 inhibited BMSC-induced bone repair in nude mice and reduced the volume of regenerated new bone. In addition, BMSCs transfected with Lv-shFibulin2 showed excellent potential for in vivo bone regeneration and repair. Compared to the WT mice, the Fibulin2 KO neonatal mice showed enhanced bone mineralization. Overall, Fibulin2 knockout contributed to increased skeletal formation.

Taken together, the results of our study shed new light on the role of Fibulin2 in bone destruction. Specifically, we found that Fibulin2 accumulated in infected bone and negatively regulated BMSC osteogenic differentiation by inactivating Notch2 signaling. This effect not only resulted in impaired osteogenesis but also triggered progressive skeletal dysfunction in the context of infected bone fracture healing. In addition to providing promising strategies for preventing osteomyelitis bone defects, the evidence generated in this study provides a theoretical basis for further research on the function of Fibulin2 in bone remodeling and Notch pathway signaling. Despite the significant findings, the limitations of the present study indicate potential areas of improvement in future research. First, bacterial components other than LPS may upregulate Fibulin2 expression, and more research is needed to explore this possibility. Second, mutagenesis experiments should be conducted to confirm the binding sites between Fibulin2 and Notch2. Finally, the mechanism by which Fibulin2 suppresses Notch2 activation and the consequences thereof remain unclear, and further study is warranted.

## Supplementary information

Supplementry figures and tables

## Data Availability

Data supporting the findings reported in this manuscript are available from the corresponding author upon reasonable request.
