# Supplementary material for: Fibulin2: a negative regulator of BMSC osteogenic differentiation in infected bone fracture healing
Source: Exp Mol Med. 2023 Feb 17;55(2):443–56. doi: 10.1038/s12276-023-00942-0 (PMC9981700; doi:10.1038/s12276-023-00942-0)
Supplement: Supplementary file 1 — Supplementry figures and tables [file 12276_2023_942_MOESM1_ESM.pdf]

Supplementary figure titles and tables

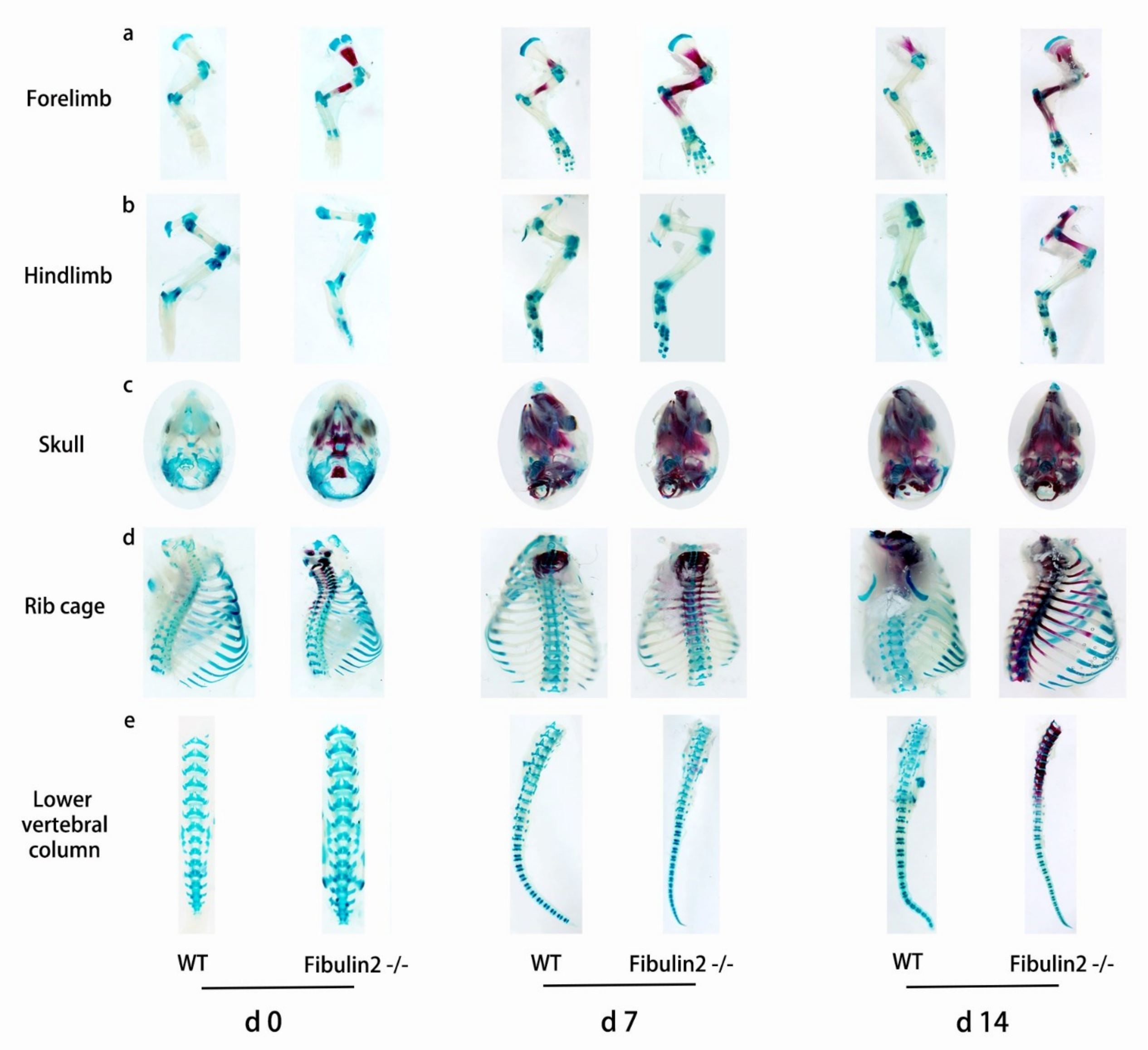

Supplementary Fig. 1 Accelerated bone formation in Fibulin2 knockout mice. Fibulin2 knockout (KO) neonatal mice and wild-type (WT) mice from the same litter were subjected to whole skeletal tissue staining with alizarin red and Alcian blue on days 0, 7 and 14. The mineralized bone was stained red, and the cartilage was stained blue. **a, b, c, d, e** Partially enlarged diagrams of

mouse structures. The forelimbs (a), skull bone (c) and rib cage (d) appeared to be mineralized in the *Fibulin2-/-* mice on days 0, 7 and 14. The mineralization rate in the hindlimbs (b) and lower vertebral column (e) was obviously higher in the *Fibulin2-/-* neonatal mouse than in the WT mice on day 14.

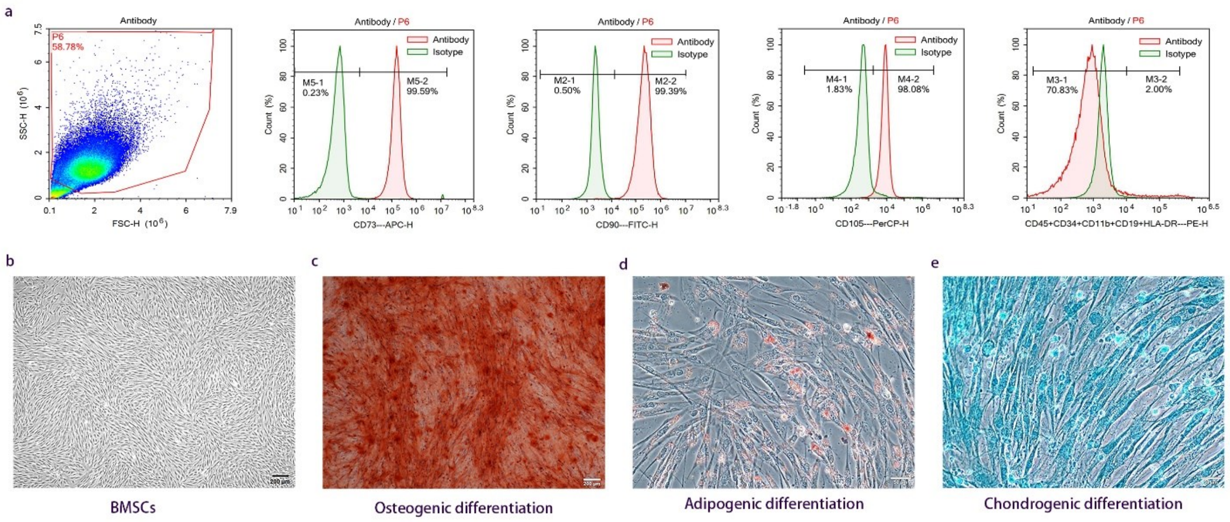

Supplementary Fig. 2 Identification of isolated BMSCs. a Flow cytometry results showed that BMSCs expressed CD105, CD90 and CD73 but not CD45, CD34, CD11b, CD19 or HLA-DR. **b** BMSCs showed plasticity in their adherence and were spindle-shaped. **c, d, e** Osteogenic, adipogenic and chondrogenic differentiation assays demonstrated that the isolated BMSCs exhibited multidifferentiation potential.

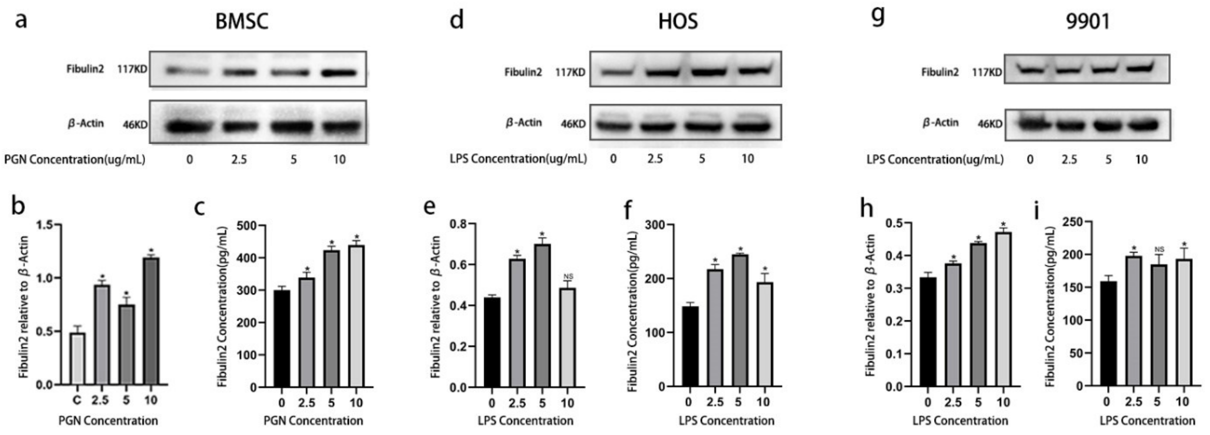

Supplementary Fig. 3 Fibulin2 expression in MSC-osteoblast lineage cells. a Fibulin2 expression in BMSCs stimulated with PGN for 24 h was detected by western blotting (WB). **b** Quantification of the WB data (* P<0.05, compared to the control, n=3). **c** Fibulin2 expression in BMSCs stimulated with PGN for 24 h was detected by ELISA (* P<0.05, compared to the control, n=3). **d** Fibulin2 expression in HOS cells stimulated with PGN for 24 h was detected by WB. **e** Quantification of the WB data (* P<0.05, compared to the control; NS, not significant; n=3). **f** Fibulin2 expression in HOS cells stimulated with PGN for 24 h was detected by ELISA (* P<0.05 compared to the control, n=3). **g** Fibulin2 expression in 9901 cells stimulated with PGN for 24 h was detected by WB. **h** Quantification of the WB data (* P<0.05, compared to the control, n=3). **i** Fibulin2 expression in 9901 cells stimulated with PGN for 24 h was detected by ELISA (* P<0.05, compared to the control; NS, not significant; n=3).

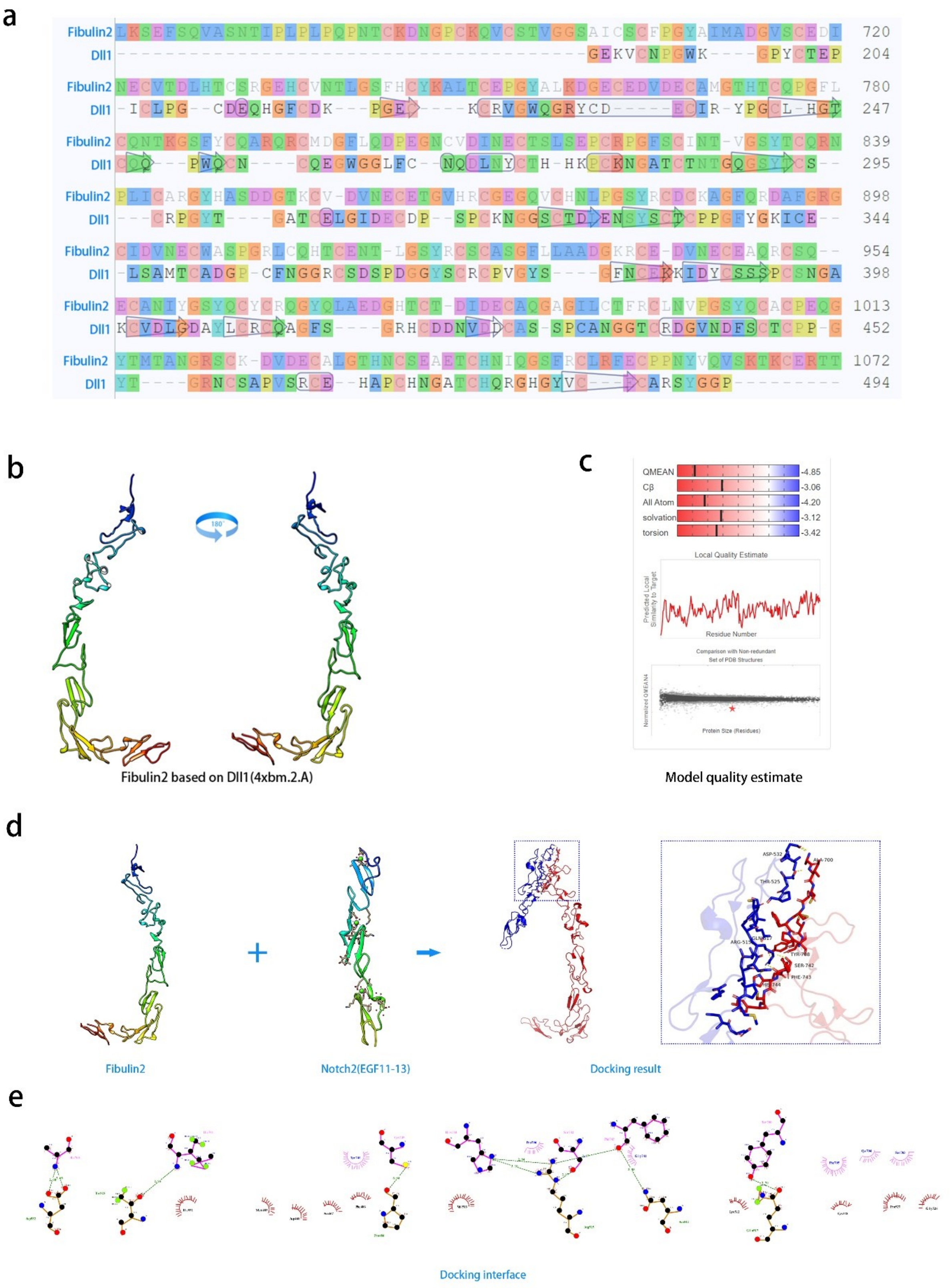

Supplementary Fig. 4 Docking between Fibulin2 and Notch2. **a** The sequence identity between Fibulin2 and Dll1 was 31.44%. **b** The spatial structure of Fibulin2, as determined by the Dll1

(4xbm.2.A) structure, was found to be L-shaped. **c** Structure quality assessment of Fibulin2. **d** The initial docking between the Fibulin2 and Notch2 structures. The docking complex was found to be J-shaped or hook-shaped. The magnified docking interface is shown in the right circled area. **e** Interacting molecules and modes of interaction related to the docking results.

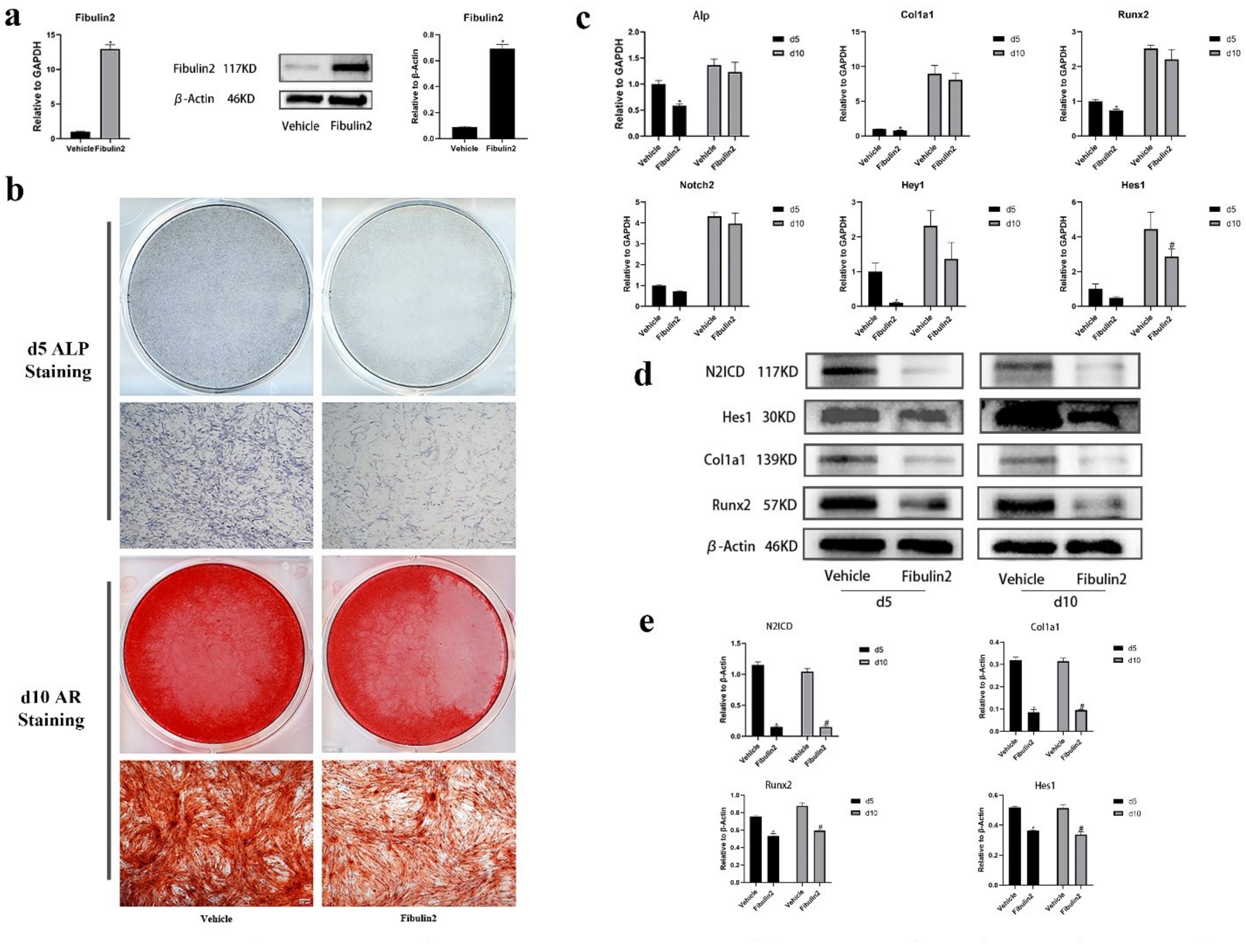

Supplementary Fig. 5 Overexpression of Fibulin2 inhibits BMSC osteogenesis by blocking Notch2 pathway activation. a Fibulin2 expression in BMSCs was analysed by RT–qPCR and western blotting (WB). Fibulin2 expression was highly upregulated in Lv-Fibulin2-infected cells compared with vehicle-infected cells (*P<0.05). **b** ALP staining and ARS on days 5 and 10 revealed

lower ALP activity and fewer mineralization nodes in the Fibulin2 group than in the vehicle group. **c** The expression of osteogenesis markers (ALP, Col1a1, and Runx2) and Notch2 pathway components (Notch2, Hey1, and Hes1) was detected by RT–qPCR. Overexpression of Fibulin2 inhibited the transcription of Alp, Col1a1, Runx2, Hey1, and Hes1 to varying degrees (*Fibulin2 group vs. vehicle group, P<0.05 on day 5; #Fibulin2 group vs. vehicle group, P<0.05 on day 10; n=3). **d** WB showed decreased expression of N2ICD, Hes1, Col1a1 and Runx2 in the Fibulin2 group. **e** Quantification of the WB data (*Fibulin2 vs. vehicle group, P<0.05 on day 5; #Fibulin2 group vs. vehicle group, P<0.05 on day 10; n=3).

Supplementary Table 1 Clinical information of the volunteers

<table>
<tr><th>Patient ID</th><th>Sex</th><th>Age</th><th>Diagnosis</th><th>Health history</th><th>Surgery</th><th>Specimen</th><th>Application of specimen</th><th>Group</th></tr>
<tr><td>1</td><td>male</td><td>55</td><td>ONFH</td><td>no</td><td>THR</td><td rowspan="10">bone</td><td rowspan="10">Proteomic studies</td><td>control</td></tr>
<tr><td>2</td><td>female</td><td>52</td><td>fracture</td><td>hypertension</td><td>ORIF</td><td>control</td></tr>
<tr><td>3</td><td>female</td><td>50</td><td>ONFH</td><td>no</td><td>THR</td><td>control</td></tr>
<tr><td>4</td><td>male</td><td>53</td><td>fracture</td><td>no</td><td>ORIF</td><td>control</td></tr>
<tr><td>5</td><td>male</td><td>45</td><td>osteomyelitis</td><td>no</td><td>debridement</td><td>osteomyelitis</td></tr>
<tr><td>6</td><td>female</td><td>34</td><td>osteomyelitis</td><td>no</td><td>debridement</td><td>osteomyelitis</td></tr>
<tr><td>7</td><td>female</td><td>63</td><td>osteomyelitis</td><td>penicillin anaphylaxis</td><td>debridement</td><td>osteomyelitis</td></tr>
<tr><td>8</td><td>male</td><td>51</td><td>After debridement of infection</td><td>no</td><td>Masquelet technique</td><td>recovered from osteomyelitis</td></tr>
<tr><td>9</td><td>male</td><td>44</td><td>After debridement of infection</td><td>no</td><td>Masquelet technique</td><td>recovered from osteomyelitis</td></tr>
<tr><td>10</td><td>female</td><td>50</td><td>After debridement of infection</td><td>no</td><td>Masquelet technique</td><td>recovered from osteomyelitis</td></tr>
<tr><td>11</td><td>male</td><td>35</td><td>ONFH</td><td>no</td><td>THR</td><td rowspan="3">bone marrow</td><td rowspan="3">Isolation of BMSCs</td><td rowspan="3">/</td></tr>
<tr><td>12</td><td>female</td><td>40</td><td>ONFH</td><td>no</td><td>THR</td></tr>
<tr><td>13</td><td>female</td><td>44</td><td>ONFH</td><td>no</td><td>THR</td></tr>
<tr><td>14</td><td>male</td><td>61</td><td>osteomyelitis</td><td>diabetes</td><td>amputation</td><td>bone</td><td></td><td>control and osteomyelitis</td></tr>
</table>

15	male	55	ONFH	no	THR	Histology and Immunohistochemistry experiment	control
16	male	68	ONFH	no	THR		
17	female	71	osteomyelitis	no	debridement		osteomyelitis
18	female	67	osteomyelitis	no	debridement		

Note: ONFH: osteonecrosis of the femoral head; THR: total hip arthroplasty; ORIF: open reduction and internal fixation.
